# Demographic representation in clinical trials for cell-based therapy

**DOI:** 10.1016/j.conctc.2021.100702

**Published:** 2021-01-07

**Authors:** Russell G. Saltzman, Dushyantha T. Jayaweera, Lina V. Caceres, Jairo A. Tovar, Mayra Vidro-Casiano, Vela Karakeshishyan, Jeanette Soto, Aisha Khan, Raul D. Mitrani, Ivonne H. Schulman, Joshua M. Hare

**Affiliations:** a Interdisciplinary Stem Cell Institute, University of Miami Miller School of Medicine, Miami, FL, USA; b Department of Medicine, University of Miami Miller School of Medicine, Miami, FL, USA

**Keywords:** Clinical trials, Cell-based therapy, Health equity, Generalizability, Diversity, Inclusion

## Abstract

Inclusion of women and minorities in clinical research is critical to fully assess the safety and efficacy of innovative therapies. With inadequate representation of demography, generalizability is impaired since pharmacokinetics and pharmacodynamics differ in these patient populations. This study was designed to analyze the voluntary participation rates of different demographic groups in cell-based therapy clinical trials conducted by the Interdisciplinary Stem Cell Institute (ISCI) at the University of Miami, Miller School of Medicine. ISCI conducted eight clinical trials between 2007 and 2017. The trials enrolled patients with ischemic and non-ischemic cardiomyopathy, idiopathic pulmonary fibrosis (IPF), aging-frailty, and Type-2 Diabetes. Participants received cell-based therapy (n = 218) or placebo (n = 33). Among the 251 participants, 29.5% were Hispanic and 20% were women. The proportion of individuals participating in each trial was compared to that of the respective disease populations attending University of Miami Health System clinics to calculate the participation to prevalence ratio (PPR). Distribution of women accurately reflected the population attending the University of Miami Health System in trials for dilated cardiomyopathy (DCM) and aging-frailty but was under-represented in others. Similarly, Hispanics and whites were accurately represented in three of the five disease fields, with Hispanics under-represented in frailty and diabetes, and whites over-represented in DCM and IPF. Black patients were accurately represented in the diabetes trial but were under-represented in all others. This study provides insight into challenges of achieving representative inclusion in research. Novel community engagement strategies are necessary to improve inclusion of women and under-represented minorities in clinical research of cell-based therapy.

## 1 Introduction

The inclusion of women and minority groups in clinical research is essential for the evaluation of therapeutic responses among different demographic groups. The overall goal of the US Public Health Service Act of 2007 [1] is to ensure that clinical trials are conducted with appropriate consideration and representation of demographic groups, including ethnicity, race, sex, and age. The University of Miami Interdisciplinary Stem Cell Institute (ISCI) [2] is actively engaging in clinical trials in regenerative medicine with novel cell-based therapies in all phases of the translational process. Pharmacokinetics and pharmacodynamics are greatly influenced by age [3,4], ancestry [5,6], and sex [[7], [8], [9]]. These variations contribute to differences in health disparities, clinical presentation, therapeutic response, and incidence of adverse effects [10]. Responses to cell-based therapies are likely beholden to similar patterns as described above, with perhaps even more variation due to the pleiotropic nature and complexity of cell therapy [11,12].

A previous meta-analysis conducted by the US Food and Drug Administration (FDA) has demonstrated that pooling individual patient data from multiple clinical trials can provide significant sex difference information, which could have gone unrecognized if individual clinical trials with under-representation of women were analyzed in isolation [13]. Enrollment of subjects with gender and race reflecting the community in clinical trials can be difficult to achieve in some cases due to strict eligibility criteria that defines the study population. Study inclusion is further hindered in the context of cell-based therapy research due to logistical complexity and safety precautions that are required.

The inclusion of diverse study populations in clinical trials is vital to understanding the role of “multi-omic” differences (i.e. genomic, transcriptomic, metabolomic, etc.) and fundamental mechanisms of disease [14], which in turn leads to improvements in safety and efficacy of the investigational therapy. Research into improving methods for participant recruitment and retention in clinical trials is an area which has been investigated thoroughly [[15], [16], [17]], with the general consensus being that recruitment strategies should be tailored to each community. One example of this is from the study by Horowitz et al. [18], which demonstrated that referrals garnered through community engagement and collaboration with community-based organizations (CBOs) outperformed that of clinician referral of under-represented minorities in a study of prediabetes.

The purpose of this study is to aggregate, summarize, and analyze demographic information collected from participants in ISCI clinical trials of regenerative medicine. These unique cohorts may offer a vital source of information about the effects of stem cell therapy on the pathophysiology of chronic disease. Identifying representativeness of trial participants can help guide future recruitment efforts among groups with need for improvement.

## 2 Methods

The studies were identified by searching the Clinicaltrials.gov web database for trials meeting the following inclusion criteria: (a) Sponsored by the Interdisciplinary Stem Cell Institute at the University of Miami, Miller School of Medicine; (b) Initiated between January 1, 2007, and December 31, 2017; and (c) Interventional study design using human mesenchymal stem cell (MSC) therapy. The query generated a list of eight clinical trials which are displayed in Table 1. The studies were in Phase I/II of development and examined safety as their primary outcome. The trial results were published in peer-reviewed journals (PROMETHEUS [19], POSEIDON [20], POSEIDON-DCM [21], TAC-HFT [22], TRIDENT [23], AETHER [24], and CRATUS [25,26]), except for one trial (ACESO) which is still ongoing but has completed participant enrollment. Individual participant data was aggregated from the secure databases of each trial and exported to statistical program for analysis, as shown in Table 2. The dataset included Trial name, Subject ID, Age at therapy, sex, race, and ethnicity. Information about study disease and route of administration was also compiled. Participants who consented but did not qualify to receive investigational therapy were excluded from the analysis.

**Table 1:** Trial overview.

Clinical Trial	NCT#	Start Date	Study Phase	Study Disease	Route of Administration	Safety Findings	Efficacy Findings
PROMETHEUS	NCT00587990	Nov-07	I/II	ICM	Epicardial Injection	No Ectopic Tissue Formation No Deaths	↑LVEF ↓Infarct Scar
TAC-HFT	NCT00768066	Aug-08	I/II	ICM	Trans-endocardial Injection (TESI)	No Ectopic Tissue Formation No Deaths No TE-SAEs No difference in AEs compared to placebo	↑QOL & 6MWT compared to placebo ↓ LV Mass compared to placebo
POSEIDON	NCT01087996	Mar-10	I/II	ICM	Trans-endocardial Injection (TESI)	No Ectopic Tissue Formation No Deaths No difference in TE-SAEs between groups	↑QOL & 6MWT in both groups ↓ LV sphericity index
TRIDENT	NCT02013674	Nov-13	II	ICM	Trans-endocardial Injection (TESI)	No Ectopic Tissue Formation No TE-SAEs No difference in AEs between groups No Immune Rejection	↑ LVEF in high dose MSC ↓ Scar size in both groups No change in QOL ↓ Inflammation
POSEIDON-DCM	NCT01392625	May-11	I/II	DCM	Trans-endocardial Injection (TESI)	No Ectopic Tissue Formation No TE-SAEs	↑ LVEF & Endothelial function in allogeneic MSC group ↓ Inflammation
AETHER	NCT02013700	Nov-13	I	IPF	Peripheral IV Infusion	No TE-SAEs Two non-study related deaths	↑ 6MWT at week 36
CRATUS	NCT02065245	Mar-14	I/II	Aging-Frailty	Peripheral IV Infusion	No TE-SAEs No Adverse Cardio-Pulmonary Events No Immune Rejection	↑QOL & 6MWT at month 6 ↓ Inflammation
ACESO	NCT02886884	Oct-17	I/II	DM2	Peripheral IV Infusion	*(Not Available)*	*(Not Available)*

Abbreviations: NCT#, National Clinical Trials identifier number; ICM, Ischemic cardiomyopathy; DCM, dilated cardiomyopathy; IPF, Idiopathic Pulmonary Fibrosis; DM2, Type 2 Diabetes Mellitus; TESI, Transendocardial Injection; IV, Intravenous; AE, adverse event; TE, treatment emergent; SAE, serious adverse event; QOL, Quality of Life; 6MWT, 6-minute walk test; MSC, Mesenchymal Stem Cell; Source: Clinicaltrials.gov. ACESO safety & efficacy data not available.

**Table 2:** Demography by study.

Clinical Trial	Age			Sex		Ethnicity		Race		
	<45	45–64	≥65	Male	Female	Hispanic	Non-Hispanic	White	Black	Other
PROMETHEUS (n = 5)	0 (0%)	5 (100%)	0 (0%)	5 (100%)	0 (0%)	5 (100%)	0 (0%)	4 (80%)	0 (0%)	1 (20%)
TAC-HFT (n = 67)	4 (5.9%)	43 (64.2%)	20 (29.9%)	63 (94%)	4 (6%)	32 (47.8%)	34 (52.2%)	60 (90%)	3 (4%)	4 (6%)
POSEIDON (n = 25)	2 (8%)	11 (44%)	12 (48%)	21 (84%)	4 (16%)	7 (28%)	18 (72%)	23 (92%)	1 (4%)	1 (4%)
TRIDENT (n = 30)	2 (6.7%)	9 (30%)	19 (63.3%)	27 (90%)	3 (10%)	5 (16.7%)	25 (83.3%)	28 (93.3%)	1 (3.3%)	1 (3.3%)
Poseidon-DCM (n = 34)	6 (17.6%)	21 (61.8%)	7 (20.6%)	24 (71%)	10 (29%)	12 (35.3%)	22 (64.7%)	30 (88%)	3 (9%)	1 (3%)
AETHER (n = 9)	0 (0%)	1 (11.1%)	8 (88.9%)	9 (100%)	0 (0%)	3 (33.3%)	6 (66.7%)	9 (100%)	0 (0%)	0 (0%)
CRATUS (n = 65)	0 (0%)	6 (9.2%)	59 (90.8%)	41 (63.1%)	24 (36.9%)	5 (7.7%)	60 (92.3%)	64 (98.5%)	0 (0%)	1 (6.3%)
ACESO (n = 16)	1 (6.3%)	7 (43.8%)	8 (50%)	11 (68.8%)	5 (31.3%)	5 (31.3%)	11 (68.8%)	12 (75%)	3 (18.8%)	1 (6.3%)
*Total (n* = *251)*	*15 (6.0%)*	*103 (41.0%)*	*133 (53.0%)*	*201 (80.1%)*	*50 (20.0%)*	*74 (29.5%)*	*176 (70.1%)*	*230 (91.6%)*	*11 (4.4%)*	*10 (4.0%)*

Values Presented as frequency (%); Other Race includes Asian, Native-American, or unknown race.

Differences in age, sex, race, and ethnicity, along with other factors within the source population for each disease can contribute to a disproportionate inclusion. In order to address the influence of this factor, information regarding the demographic composition of the various disease populations within the University of Miami Health System (UHealth) was also obtained. Since the University of Miami Health System was the enrolling site for all eight clinical trials, the UHealth system database was determined to be the most accurate source of data to estimate demography of the various disease populations. The background rate was determined for each disease: ischemic cardiomyopathy (ICM), dilated cardiomyopathy (DCM), idiopathic pulmonary fibrosis (IPF), aging-frailty, and type 2 diabetes mellitus (DM2). The University Research Informatics Data Environment (URIDE) [27] was used to generate the query reports from UChart (EPIC Systems Corp., Verona, WI, USA), the University of Miami Health System (UHealth) patient database. Population demographics (sex, race, and ethnicity) summary data was extracted from the database for all patients seen by the UHealth system between the years 2007–2017 with the specific disease indications. The source populations for each of the disease indications are shown in Table 3. We used the following ICD10 codes to obtain the source population estimates from the UChart database. The ICD-10 code *I25.5* was used to search for patients with ICM, *I42.*0 for patients with DCM, *R54* for Aging-Frailty, *J84.112* for Idiopathic Pulmonary Fibrosis and *E11.9* for Type 2 Diabetes Mellitus.

**Table 3:** Source population prevalence estimates.

Disease	ICD 10 Code	Sex		Ethnicity			Race				
		Male	Female	Hispanic	Non-Hispanic	Unknown	White	Black	Asian	Native American	Other
ICM (N = 1422)	I25.5	83.61%	16.39%	41.21%	56.61%	2.18%	77.71%	10.83%	0.77%	0.07%	10.62%
DCM (N = 1010)	I42.0	63.56%	36.44%	37.03%	61.09%	1.88%	69.70%	20.89%	1.09%	0.10%	8.22%
IPF (N = 279)	J84.112	70.25%	29.75%	35.13%	60.22%	4.66%	78.85%	5.02%	0.72%	0.00%	15.41%
Frailty (N = 265)	R54	56.98%	43.02%	12.45%	44.15%	1.51%	87.92%	3.77%	1.13%	0.00%	7.17%
DM2 (N = 42,308)	E11.9	53.09%	46.91%	42.72%	52.88%	4.40%	63.79%	19.91%	1.51%	0.21%	14.57%

Abbreviations: ICD-10, International Statistical Classification of Diseases and Related Health Problems-10th revision; ICM, Ischemic cardiomyopathy; DCM, dilated cardiomyopathy; IPF, Idiopathic Pulmonary Fibrosis; DM2, Type 2 Diabetes Mellitus; Source: URIDE, UHealth System - University of Miami.

The proportion of individuals participating in trials was compared to that of the respective disease populations attending University of Miami health system clinics in order to compute the participation to prevalence ratio (PPR) for each demographic subgroup. **PPR** = **Proportion among trial participants**÷ **Proportion among Disease Population.** The result is expressed as a ratio, as described by Eshara et al. [28]. PPR of 1.0 indicates that the level of inclusion in the trials is equal to the proportion of that group in the intended patient population of the study disease. A ratio between 0.8 and 1.2 indicates that the proportion of that subgroup enrolled into the trials are similar to the proportion in the disease population. A PPR <0.8 signifies under-representation of the group, and a PPR >1.2 indicates an over-representation of the subgroup [28]. The analysis was generated using SAS software, Version 9.4. (SAS Institute Inc., Cary, NC, USA).

## 3 Results

**Fig. 1:**
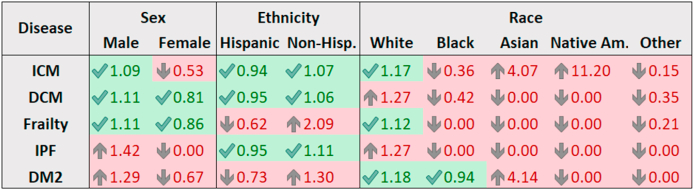
Comparison of PPR by subgroups and study disease. Abbreviations: PPR, Participation to Prevalence Ratio; ICM, Ischemic cardiomyopathy; DCM, dilated cardiomyopathy; IPF, Idiopathic Pulmonary Fibrosis; DM2, Type 2 Diabetes Mellitus; PPR Calculation: PPR = Proportion among trial participants ÷ Proportion among Disease Population. PPR Interpretation: <0.8 = Under-Representation, 0.8-1.2 = Adequate Representation, >1.2 = Over-Representation.

The clinical trials under investigation in this study are presented in Table 1. Among the 251 subjects, 50 were women (20%) and 201 were men (80%). High rates of participation among women were found in some but not all studies. For example, the trials for DCM, DM2, and aging-frailty enrolled women at a rate higher than the overall average with 29%, 31.3%, and 36.9% respectively. When comparing between study sample representation (Table 2) and that of the various source populations (Table 3), it becomes evident that women were under-represented in the trials for ICM, IPF, and DM2, but were adequately represented in DCM and aging-frailty (Fig. 1). Men were suitably represented in the trials for ICM, DCM, and Frailty, but were over-represented in trials of DM2 and IPF, also displayed in Fig. 1. The racial and ethnic composition across the various trials shows that 230 (91.6%) participants were white, 11 (4.4%) were black, and 10 (4%) were of Asian, Native American, or unknown race. Hispanic patients comprised 29% of all participants. The primary recruitment source for these trials was through physician referral and patient self-referral. All 5 participants treated in the PROMETHEUS study were white Hispanic males. The TAC-HFT trial had the next highest proportion of Hispanic participants with 48%. There was a total of 30 participants across all eight trials which were considered screen failures or had withdrawn consent before receiving study therapy and were excluded from the PPR analyses calculations. This group was comprised of 8 Hispanic individuals, 21 Non-Hispanic individuals, and one of unknown ethnicity. As shown in Fig. 1, inclusion of Hispanics was similar to that of the source population of ICM, DCM, and IPF, but were under-represented in studies of Aging-frailty and DM2. Black participants were adequately represented in the study of DM2 but were under-represented in all other disease groups. Asians and Native Americans make up a small percentage to the UHealth Database, therefore the studies of ICM and DM2 that had Asian or Native American participants showed this as an over-representation of these groups.

## 4 Discussion

Cell based therapy has the potential to be used as a novel treatment option for a variety of chronic diseases and conditions [29]. In this study we compared the trial enrollment rates to the prevalence of the disease found in the source population. The fundamental premise is that sex, race, and ethnicity of the study participant matters, and different demographic groups can behave and react differently. Data on women and under-represented minorities is critical to understand the safety and efficacy profiles of a novel therapy.

**Fig. 2:**
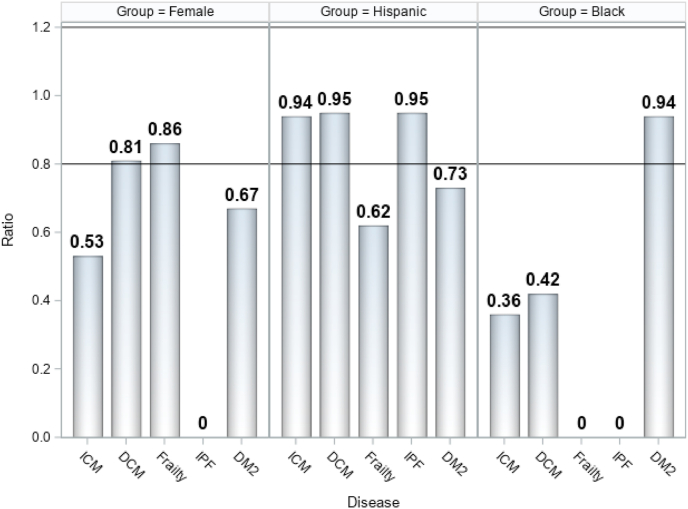
Participation to prevalence ratio (PPR) for historically under-represented minorities. Abbreviations: PPR, Participation to Prevalence Ratio; ICM, Ischemic cardiomyopathy; DCM, dilated cardiomyopathy; IPF, Idiopathic Pulmonary Fibrosis; DM2, Type 2 Diabetes Mellitus; PPR Calculation: PPR = Proportion among trial participants ÷ Proportion among Disease Population. PPR Interpretation: <0.8 = Under-Representation, 0.8-1.2 = Adequate Representation, >1.2 = Over-Representation.

As displayed in Fig. 2, our findings show that women had satisfactory representation in the trials of DCM and aging-frailty, but were under-represented in DM2, IPF, and ICM. Hispanic populations were accurately represented in trials for ICM, DCM, and IPF, however were under-represented in studies for aging-frailty and DM2. Black participants were under-represented across all trials except for DM2. The results of this analysis support the idea that there are differences in enrollment between demographic groups in our cell therapy clinical trials and in future, larger phase 2–4 studies this must be rectified.

The challenges to enrollment of women and under-represented minorities into clinical trials are well documented. Liu et al. provides an overview of the systematic barriers to women's participation in research from a historical overview, and how this has been addressed through policy changes and initiatives [30]. Members of minority groups may be less likely to participate in trials due to logistical challenges, financial burden, and/or lingering distrust from historical victimization in medical experiments [31]. Heller et al. describes strategies to overcome these barriers to clinical trial enrollment affecting people of color [32].

Based on publicly available FDA reviews of women enrolled in late-phase pivotal-trials supporting 36 drug approvals from 2005 to 2015, women were well represented in trials of drugs for hypertension and atrial fibrillation and over-represented for pulmonary arterial hypertension. A recent phase 1 study of allogeneic MSCs for anthracycline-induced cardiomyopathy (SENECA; NCT02509156) also had a high representation (68%) of women [33]. Representation of women fell below a PPR of 0.8 for trials in heart failure, coronary artery disease, and acute coronary syndrome, with minimal sex differences in drug efficacy and observed safety profiles [34]. This may adversely impact the ability to assess biologic effects of the drugs for these conditions.

Also shown in Fig. 2, the PPR for Hispanics with ICM was 0.94, which is a close approximation to the source population. Regarding the breakdown of race with ICM, whites were slightly over-represented (1.1), Blacks were under-represented (0.36), and Asians (4.07) and Native-Americans (11.2) were highly over-represented. Over-representation of Asians and Native-Americans should not be considered as noteworthy since the sample size was small with low prevalence of the disease in the source population.

The other disease populations were also examined for representativeness, which indicated that recruitment of women into the studies was adequate but still offers room for improvement. Among the ICM trials, the PPR for inclusion women was 0.52, indicating under-representation. Women enrolled into the DCM trial at a ratio of 0.80 and in aging-frailty at 0.87. Although the ratios are not exactly proportional, enrollment of women in studies of these two diseases were within 20% of the optimum inclusion rate and is considered adequate/similar.

Participation among women with DCM and aging-frailty was found to be proportional to the disease prevalence, but women were under-represented in ICM, IPF, and DM2. No women participated in the IPF trial, however the prevalence of IPF among women in the source population (30%) was lower than all other diseases studied. Men were slightly over-represented in the trials for DM2 (1.29) and IPF (1.42), however adequate enrollment of men was observed in all other disease fields.

The US Code of Federal Regulations [1] stipulates that women and members of minority groups must be included and reported in National Institutes of Health (NIH) supported research, regardless of developmental phase. The main goal of this regulation is to ensure that research findings are generalizable to the entire population. All NIH-funded clinical research studies must address plans to include these historically under-represented groups during the design of the research, and any exclusions based on sex, gender, race, or ethnicity must be justified on a scientific or ethical basis. Although the FDA does not possess the regulatory authority to require specific levels of minority inclusion in clinical trials, diversity is strongly encouraged. The 21st Century Cures Act [35] requires study investigators to submit a stratified analysis by sex/gender and race and/or ethnicity to clinicaltrials.gov to monitor adherence to this regulation [36]. Accordingly, demographic reporting has become part of the evaluation criteria in grant applications and journal peer-reviews. Although these regulations are only applicable to NIH-funded projects, they encourage all researchers to consider sex and ancestry as biological variables that should be factored into research design, analyses, and reporting in human as well as animal studies. Although this concept traces back millennia, as Hippocrates was one of the first to describe differences in disease patterns between men and women [37], inclusion of women and under-represented minorities has become an important aspect to be considered throughout the entire process of conducting clinical research in modern medicine.

Other important reasons to strive for accurate demographic representation in clinical trials are to ensure generalizability of the findings, work to eliminate health disparities among underserved communities by improving access to novel therapies, and to better understand disease pathophysiology among various racial and ethnic groups. A recent meta-analysis of cell therapy trials explored the safety profile of MSCs through a systematic literature review [38]. The study included safety data from 55 randomized controlled trials across eight different disease fields and did not detect any significant associations between MSC therapy and incidence of serious adverse events. Future research in this area could explore the differences in safety outcomes based on demography.

Apart from the DM2 and frailty trials, the Hispanic population was accurately represented within all other diseases. The representation of Black or African American patients was underwhelming among the study diseases, with the exception of the DM2 study (0.94). This information provides us with insight into the challenges of achieving representative enrollment of participants for cell therapy clinical trials. Further efforts must be made to recruit diverse populations into cell therapy clinical trials. New methods are needed to engage with stakeholders and community members to improve enrollment of women, Black and Hispanic patients.

The main strength of this study is the relevance to public health. Cell based therapy is a novel intervention with potential applications to a broad spectrum of disease fields, and a focus on inclusion of women and under-represented minorities will improve the quality and generalizability of the information gained. Limitations to this research are also present, such as the absence of information about recruitment/referral source or motivations for study participation. Information regarding community engagement at the trial design stages to evaluate the community input into study design was also not available. Source population prevalence data was obtained using ICD-10 codes for patients at a single health system and may not be an accurate representation of disease prevalence in the general population of this or other regions and time-periods. There are limitations in the estimation of the PPR used in this study and others. The values used to determine the proportion of Hispanic patients in the source population were compiled from electronic medical records and errors in medical coding or diagnosis may affect the final determination. Furthermore, our ability to perform meta-analyses is limited by the availability of data from early phase, industry sponsored, clinical trials. Future research may address these issues using advanced statistical methods.

In summary, women had proportional representation in some (DCM and aging-frailty) clinical trials, but not other (DM2, IPF, and ICM) clinical trials. People of Hispanic heritage were accurately represented in studies of ICM, DCM, and IPF, but not with aging-frailty or DM2. People of Black or African decent were under-represented across all trials except for DM2. These findings highlight the challenges of enrolling representative populations in a large single-center cell-based therapy research unit. Early community engagement with bidirectional communications will be most helpful to achieve accurate inclusion of women and under-represented minorities in clinical research of cell-based therapy.

## Disclosures

Joshua M. Hare reports having a patent for cardiac cell-based therapy, holds equity in Vestion Inc., and maintains a professional relationship with Vestion Inc. as a consultant, member of the Board of Directors, and Scientific Advisory Board. Dr. Joshua Hare is the Chief Scientific Officer, a compensated consultant, board member, and holds equity in Longeveron. Dr. Hare is also the co-inventor of intellectual property licensed to Longeveron. These relationships are reported to the University of Miami, and an appropriate management plan has been instituted. Ivonne H. Schulman has no disclosures and contributed to this manuscript in her personal capacity. The opinions expressed in this article are the author's own and do not reflect the view of the National Institutes of Health, the Department of Health and Human Services, or the United States government. Aisha Khan discloses a relationship with AssureImmune Cord Blood Bank and Aceso Therapeutic that includes equity. Russell G. Saltzman, Lina V. Caceres, Jairo A. Tovar, Mayra Vidro-Casiano, Vela Karakeshishyan, Jeanette Soto, Dushyantha T. Jayaweera, and Raul D. Mitrani have nothing to disclose.
